# Current Advances and Future Prospects for Molecular Research for Agronomically Important Traits in Rice

**DOI:** 10.3390/ijms23147531

**Published:** 2022-07-07

**Authors:** Kiyosumi Hori, Matthew Shenton

**Affiliations:** National Agriculture and Food Research Organization, Institute of Crop Science, Tsukuba 305-8518, Japan

Rice (*Oryza sativa* L.) is the most important food crop in the world, being a staple food for more than half of the world’s population. The significance of rice as a crop and as a model species promoted overseas collaborations leading to the formation of the International Rice Genome Sequencing Project, and rice became the first crop genome to be sequenced in 2004 [1]. After the release of the first complete rice genome of ‘Nipponbare’, researchers have constructed several reference genomes for other rice cultivars including ‘93-11’, ‘IR64’, ‘Zhenshan 97’, and ‘Minghui 63’, and collected large sets of whole-genome sequence data comprising more than 3000 rice cultivars distributed worldwide [2,3]. These fundamental genomic data resources are easily accessed via public databases such as the MSU Rice Annotation Project (http://rice.uga.edu/, accessed on 4 July 2022), the RAP-DB (https://rapdb.dna.affrc.go.jp/, accessed on 4 July 2022), and the TASUKE+ (https://tasuke-plus.dna.affrc.go.jp/, accessed on 4 July 2022) [4,5,6]. Recent advances in molecular genomics and genetics tools are represented in other databases including the RiceXpro transcriptome atlas (https://ricexpro.dna.affrc.go.jp/, accessed on 4 July 2022) and the Rice Expression Database (RED) (http://expression.ic4r.org/, accessed on 4 July 2022) [7]. Mutant populations can be found at the RMD (http://rmd.ncpgr.cn, accessed on 4 July 2022) and the KitBase (https://kitbase.ucdavis.edu/, accessed on 4 July 2022) [8]. Molecular research tools are well-established in rice, and are increasing our knowledge about genetic factors at QTLs and responsible genes controlling agronomic traits. Analysis of rice genome sequences has shown that there are more than 37,000 genes in the ~400 Mbp rice genome [1,4,5]. However, as of 2022, there are only about 3000 genes whose molecular functions are characterized in detail, e.g., the developmental stages and tissues where they are expressed from germination to harvest, and how they are co-expressed with other genes and proteins [9]. The individual molecular functions of most of the remaining genes are still unknown so far. Therefore, continuing many research efforts in gene functional analysis is necessary to elucidate the genetic networks and molecular mechanisms controlling agronomically important traits.

Recent improvements in living standards have increased the worldwide demand for high-yielding and high-quality rice cultivars. To develop novel cultivars with superior agronomic performance, we need to understand the molecular basis of agronomically important traits related to grain yield, grain quality, disease resistance, and abiotic-stress tolerance. Molecular biology techniques can reveal the complex mechanisms involved in the control of these agronomic traits [10,11]. After editing the previous Special Issue in the International Journal of Molecular Science [9], we have continued to collect recent significant studies that identified genetic factors and revealed their molecular contributions to rice agronomic traits in the Special Issue “Molecular Research in Rice: Agronomically Important Traits 2.0”.

Rice grain yield consists of four main components: the number of panicles per plant, the number of grains per panicle, the percentage of ripening grains, and 1000-grain weight. Changes in panicle architecture have been associated with improved grain yield. In rice, panicle architecture is mainly determined by the spikelet and branch arrangement. Spikelets and branches are initiated and developed from inflorescence meristems. Li et al. showed that the *Verticillate Primary Branch 1* (*VPB1*) gene is preferentially expressed in the inflorescence meristem and is associated with control of primary branch arrangement [12]. The *VPB1* gene encodes a BELL-like homeodomain (BLH) protein, which alters the expression of other genes involved in inflorescence meristem identity and hormone-signaling pathways by binding to the promoter region of the *OsBOP1* gene. Yin et al. summarized recent advances concerning genes involved in controlling the grain number per panicle (GNPP) in rice [13]. They collected a total of 36 genes affecting GNPP, such as *DEP1*, *IPA1*, *APO1*, *TAW1*, and *SP1*, and classified these GNPP-related genes based on their molecular functions such as positive or negative regulation of GNPP by rachis branch development, phase transition from rachis branch meristem to spikelet meristem, and spikelet specialization. Park et al. focused on grain shape and grain weight. They detected a large-genetic-effect QTL on rice chromosome 1, and estimated that the candidate gene of this QTL is the *OsBRKq1* gene [14]. The *OsBRKq1* gene encodes brassinosteroid leucine-rich repeat-receptor kinase protein, is expressed at the spikelet differentiation stage, and positively affects grain size. Usman et al. created long-grain mutant lines of the *GS3* gene by using CRISPR/Cas9-mediated genome editing [15]. The genome-edited lines showed a grain length and 1000-grain weight increased by about 25%. These novel alleles of the *GS3* gene altered the expression of other genes including those annotated with cysteine synthase, cysteine proteinase inhibitor, vacuolar protein sorting-associated, ubiquitin, and DNA ligase GO (gene ontology) terms.

The understanding of the genetic basis for various abiotic stress resistance and tolerance is important for rice cultivation, because these stress conditions cause significant decreases in yield and grain quality. Bang et al. characterized the *Rice Chloroplast RNA-binding Protein 1* (*OsCRP1*) gene, which is essential for the stabilization of RNAs from the NAD(P)H dehydrogenase complex in rice chloroplast [16]. They clearly indicated that the *OsCRP1*-overexpressing lines showed higher cyclic electron transport activity and elevated ATP levels for photosynthesis, compared with wild-type plants. Additionally, the *OsCRP1*-overexpressing lines significantly enhanced drought- and cold-stress tolerance. Huang et al. reported that the *Rice WRKY Transcription Factor 55* (*OsWRKY55*) gene is involved both in drought responses and in plant growth regulation [17]. They created *OsWRKY55*-overexpression lines, and revealed that the overexpression lines showed faster water loss and a greater accumulation of hydrogen peroxide (H_2_O_2_) and superoxide radicals (O_2_^−^) in leaves under drought-stress conditions, and consequently decreased drought resistance compared with wild-type plants. They revealed protein–protein interactions of the OsWRKY55 protein with mitogen-activated protein kinases (MAPKs) OsMPK7, OsMPK9, OsMPK20-1, and OsMPK20-4 that could be induced by drought conditions, and showed binding activity to the promoter region of the *OsAP2-39* gene that controls cell size and plant height. Wu et al. used marker-assisted selection to introduce the *Sub1A* gene and develop breeding lines with strong submergence tolerance [18]. The developed lines exhibited desirable agronomic traits including high grain yield and quality, and showed strong drought and submergence tolerance. Abiotic stresses induced by high or low temperatures are also an important issue for rice cultivation because they cause significant decreases in grain yield and quality. Hori et al. detected two quantitative trait loci (QTLs) for the determination of flowering time according to seasonal temperature conditions [19]. The *Hd16* gene is one of the genetic factors associated with robustness of flowering time to environmental fluctuations. These research efforts are helpful in dissecting the genetic basis of stress resistance and tolerance in future breeding programs.

Nitrogen is an essential macronutrient that plays a critical role in the growth and development of rice plants. Improvement in nitrogen use efficiency is required to achieve the Sustainable Development Goals in agriculture and crop production. Kabange et al. showed that the nitrate reductase (NR) gene of *OsNR2* and nitrate transporter (NRT) genes of *OsNIA1* and *OsNIA2* were differentially expressed between the root, leaf, and stem in rice [20]. The results in their study suggested that the *NR* and *NIR* genes had tissue-specific molecular functions in response to potassium chlorate (KClO_3_) and the reduction activities of the ammonium transporters and glutamate synthase. Sato et al. developed a novel three-dimensional (3D)-imaging method with single-cell resolution [21]. The authors successfully captured shoot, floret, and root apical meristems at cellular resolution, and revealed the 3D distribution of auxin-signaling pathway proteins in the columella, quiescent center, and multiple cell rows of the root apical meristem.

The significant studies mentioned here demonstrate the importance of the research community in understanding and explaining the molecular genetic basis of agronomically important traits in rice. To develop novel rice cultivars showing good agronomic performance and strong climate resilience in the future, we have to identify further important genes, elucidate their molecular functions, and design desirable genotypes based on individual and interaction effects of those genes. In addition to the traits described in the previous and present Special Issues, there are many other agronomically important traits that should be explored using molecular genetics and biological research. In addition, the wide genetic diversity that exists in rice genetic resources including wild rice species (e.g., *Oryza rufipogon* Griff., *Oryza longistaminata* Chev. et Roehr.) has not been fully exploited in genetic analysis and breeding programs. Although several disease resistance genes have been introduced from wild species into rice cultivars [22], wild rice species also possess strong abiotic-stress resistance such as drought resistance, submergence tolerance, and anaerobic resistance. Some varieties exhibit early-morning flowering, thus avoiding anthesis at high temperature conditions closer to midday [23]. Further exploitation of this wide genetic diversity should be a priority. The sharing of research results among researchers is necessary to address problems of food security against a background of increasing demand around the world.

Following the construction of the first rice genome reference sequence, whole-genome reference sequences have been published in other crop species such as wheat, barley, maize, and soybean, as well as in vegetables, fruit trees, and ornamental plants [24]. Comparative genome sequence and homology analysis can identify highly conserved gene families among various crop species such as those involved in plant hormone synthesis and those controlling tissue development and stress responses. The gene-regulatory network for flowering time determination by florigens is also well-conserved among plant species [25], as are the polyphenol biosynthesis pathways associated with the coloring of edible parts. These pathways are targets for increasing functional food components with antioxidative effects, and providing responsiveness to ambient temperatures in almost all cereal, vegetable, and fruit tree crops. Abundant genome sequence information and annotated gene lists in rice will facilitate the improvement in various agronomic traits, not only in rice, but also in other monocot cereal crops, as well as in other dicotyledonous crop species.
